# Multiple functions of non-hypophysiotropic gonadotropin releasing hormone neurons in vertebrates

**DOI:** 10.1186/s40851-019-0138-y

**Published:** 2019-07-22

**Authors:** Chie Umatani, Yoshitaka Oka

**Affiliations:** Department of Biological Sciences, Graduate School of Science, the University of Tokyo, Tokyo, 113-0033 Japan

**Keywords:** Non-hypophysiotropic GnRH, Vertebrate, Neuromodulation, Neuropeptide

## Abstract

Gonadotropin releasing hormone (GnRH) is a hypophysiotropic hormone that is generally thought to be important for reproduction. This hormone is produced by hypothalamic GnRH neurons and stimulates the secretion of gonadotropins. On the other hand, vertebrates also have non-hypophysiotropic GnRH peptides, which are produced by extrahypothalamic GnRH neurons. They are mainly located in the terminal nerve, midbrain tegmentum, trigeminal nerve, and spinal cord (sympathetic preganglionic nerves). In vertebrates, there are typically three *gnrh* paralogues (*gnrh1*, *gnrh2*, *gnrh3*). GnRH-expression in the non-hypophysiotropic neurons (*gnrh1* or *gnrh3* in the terminal nerve and the trigeminal nerve*, gnrh2* in the midbrain tegmentum) occurs from the early developmental stages. Recent studies have suggested that non-hypophysiotropic GnRH neurons play various functional roles. Here, we summarize their anatomical/physiological properties and discuss their possible functions, focusing on studies in vertebrates. GnRH neurons in the terminal nerve show different spontaneous firing properties during the developmental stages. These neurons in adulthood show regular pacemaker firing, and it has been suggested that these neurons show neuromodulatory function related to the regulation of behavioral motivation, etc. In addition to their recognized role in neuromodulation in adult, in juvenile fish, these neurons, which show more frequent burst firing than in adults, are suggested to have novel functions. GnRH neurons in the midbrain tegmentum show regular pacemaker firing similar to that of the adult terminal nerve and are suggested to be involved in modulations of feeding (teleosts) or nutrition-related sexual behaviors (musk shrew). GnRH neurons in the trigeminal nerve are suggested to be involved in nociception and chemosensory avoidance, although the literature on their electrophysiological properties is limited. Sympathetic preganglionic cells in the spinal cord were first reported as peptidergic modulatory neurons releasing GnRH with a putative function in coordinating interaction between vasomotor and exocrine outflow in the sympathetic nervous system. The functional role of non-hypophysiotropic GnRH neurons may thus be in the global modulation of neural circuits in a manner dependent on internal conditions or the external environment.

## Introduction

**Fig. 1:**
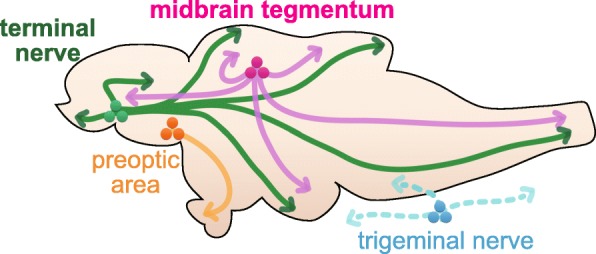
Schematic illustration of both hypophysiotropic and non-hypophysiotropic GnRH neurons in a teleost brain. Hypophysiotropic neurons: Orange, GnRH neurons in the preoptic area. Non-hypophysiotropic neurons: Green, GnRH neurons in the terminal nerve, Pink, GnRH neurons in the midbrain tegmentum, Blue, GnRH neurons in the trigeminal nerve. Solid lines schematically illustrate axons or dendrites of GnRH neurons in each brain region, and dotted lines show likely projections

Gonadotropin releasing hormone (GnRH) was first identified as a peptide that is released from hypothalamic neurons at their axon terminal in the pituitary or the median eminence, and which promotes the secretion of luteinizing hormone [1–3]. It is now known that most vertebrates have three paralogous genes for GnRH (*gnrh1–3*). In the brain, *gnrh1* is expressed in the hypothalamus/preoptic area, *gnrh2* in the midbrain tegmentum, and *gnrh3* in the terminal nerve neurons [4–6] (Fig. 1). GnRH-expressing neurons in the midbrain tegmentum and the terminal nerve project their axons broadly in the brain, except the pituitary. It has also been reported that GnRH2 and GnRH3 immuno-reactive fibers project as far as to the spinal cord [5, 7, 8], and that *gnrh3*-expressing neurons can be found in the trigeminal nerve [7, 9]. It should be noted that some species have lost one or two *gnrh* paralogues, and in species that have lost either *gnrh1* or *gnrh3*, the remaining one is expressed compensatorily in the brain region, where the lost paralogue had originally been expressed [8, 10–13] (functional compensation for the loss of a GnRH paralogue [4]). For example, zebrafish lost *gnrh1*, and zebrafish *gnrh3* is expressed not only in the terminal nerve, but also in the preoptic area. Because all GnRH subtypes can activate all subtypes of GnRH receptors [14, 15], it is functionally important for animals that neurons projecting to a specific brain region (pituitary, median eminence, sensory processing region, etc.) express one of the three *gnrh* paralogues.

Unlike hypophysiotropic GnRH, it has been suggested that GnRH expressed in the terminal nerve and midbrain tegmentum does not regulate reproductive functions, but rather modulates neural activities in regions to which GnRH neurons project. This function, which is now called “neuromodulation,” was first reported in peptidergic modulation of sympathetic ganglion neurons by GnRH [16]. This GnRH has been suggested to be GnRH2; details follow in the section *Sympathetic preganglionic neurons*. In addition to earlier studies on the neuromodulatory functions of GnRH in the sympathetic nervous system, recent studies of neuromodulation in in vitro brain preparations have shown that non-hypophysiotropic GnRHs modulate synaptic transmission in various sensory processing pathways [17–20]. In contrast, the functions of non-hypophysiotropic GnRH neurons at the organismal level remain the subject of debate.

In this review, we summarize recent studies on the anatomy and physiology of the non-hypophysiotropic GnRH neurons and discuss their possible functions. Because the number of previous studies on non-hypophysiotropic GnRH neurons using birds and mammals is much smaller than that using teleosts, we will discuss research mainly using teleosts. In addition, for the discussion of morphology of non-hypophysiotropic GnRH neurons, we only cite literature that explicitly describes the specificity of GnRH-antibody.

### GnRH neurons in the terminal nerve

#### Anatomy

**Fig. 2:**
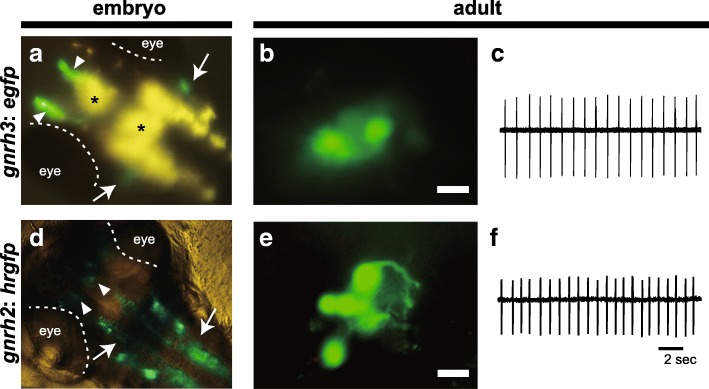
Anatomical and electrophysiological characteristics of GnRH neurons in the terminal nerve and the midbrain tegmentum (**a**) A ventral view of the brain of *gnrh3*:*egfp* medaka embryo. GFP-expressing neurons in the terminal nerve (arrowhead) and the trigeminal nerve (arrow). **b** Conventional fluorescence microscopic view of the cluster of terminal nerve neurons. Bar, 20 μm. Embryos of (**a**) and (**d**) are 4~5 dpf. Yellow autofluorescence (*) originates from chromatophores of medaka body. (**c**) Spontaneous pacemaker activity of adult medaka (12.5 weeks after fertilization) recorded by a targeted on-cell patch clamp recording. (**d**) Ventral view of *gnrh2*:*hrgfp* medaka embryo. The arrowhead indicates GnRH neurons in the midbrain tegmentum (arrowheads). Dense GFP-signals (arrow) are also observed in the spinal cord. (**e**) Enlarged view of the cell bodies, similar to B. Bar, 20 μm. (**f**) Spontaneous pacemaker activity from a *gnrh2*:*hrgfp* neuron in adult medaka. **e** & **f**: modified from Kanda et al., 2010 [22]

The terminal nerve, which has been describes as the cranial nerve zero, contains neurons expressing *gnrh1* or *gnrh3* from the early developmental stages. The neurons of the terminal nerve originate from the olfactory placode [7, 9, 21] and migrate to regions near the olfactory bulb (Fig. 2a). This migration is controlled by chemokine signaling (CXCR4-CXCL12/SDF1 signaling) [23, 24]. In most teleost species, GnRH neurons in the terminal nerve form a cluster of cell bodies (Fig. 2b). Their fibers project broadly in the brain, except the pituitary [13, 25]. In teleosts, this projection pattern is completed before hatching [9, 26].

#### Physiology

**Fig. 3:**
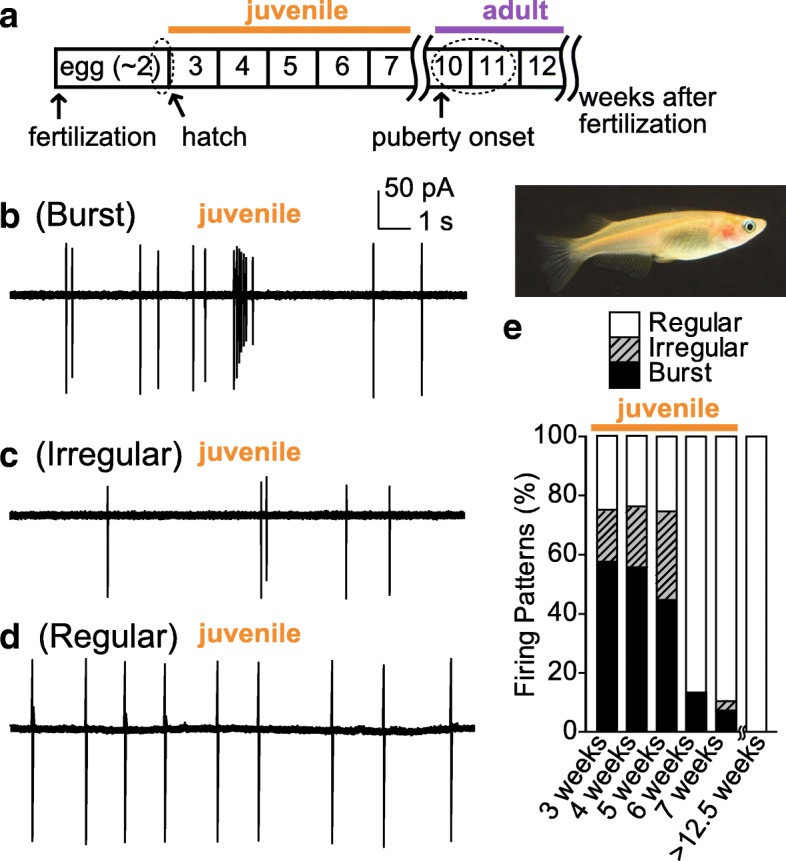
Firing patterns of the terminal nerve GnRH neurons in juvenile medaka (**a**) A scheme of post-hatching development of medaka. (**b–e**) Representative firing patterns in medaka. The firing patterns in B and C were recorded from medaka 4 weeks after fertilization, and that in D from medaka 7 weeks after fertilization. The time and current scales shown in the upper right corner of B apply to B–D. (**e**) Categorized firing patterns of TN-GnRH3 neurons in one typical fish for each stage. We used the average value for each pattern. The classification of the firing pattern is as follows: burst firing, consecutive spikes more than three at > 3 Hz (firing frequency in adult medaka is usually lower than 2 Hz) and interburst interval longer than 1 second (longer than that of typical TN-GnRH3 neurons in adults); regular firing, coefficient of variation of the interburst interval < 0.65 (the coefficient of variation is defined as the ratio of the standard deviation to the mean interburst interval); irregular firing, others. Juvenile: 3 weeks after fertilization, nine fish; 4 weeks, nine fish; 5 weeks, seven fish; 6 weeks, eight fish;7 weeks, eight fish. Adult: > 12.5 weeks, four fish. These figures were modified from Umatani and Oka, 2018 [27]

The firing activities of GnRH neurons in the terminal nerve have been studied by using a whole brain in vitro preparation of teleosts [25]. Because the GnRH neurons of the terminal nerve of fish used in these experiments express *gnrh3*, we call GnRH neurons in the terminal nerve as “TN-GnRH3 neurons” in this section. The firing pattern of adult TN-GnRH3 neurons is usually regular (1~2 Hz in medaka, 3~4 Hz in dwarf gourami) (Fig. 2c) [25, 27]. In the adult dwarf gourami, only a few percentages of TN-GnRH3 neurons show irregular or burst firing. On the other hand, in juvenile fish, these neurons show burst firing more frequently than in adulthood [27, 28] (more than 50% of them show burst firing in medaka, Fig. 3, see *Functions*). Although the regular pacemaker firing is generated by intrinsic ion channels [25], the other firing patterns are probably caused by synaptic or auto−/paracrine inputs. Electrophysiological studies on neural inputs to the TN-GnRH3 neurons have suggested the followings: glutamatergic [29] and GABAergic [30] inputs upregulate firings of these neurons, and cholinergic synaptic inputs [31] and inputs containing RFamide-related peptide [32] downregulate them. Especially, glutamatergic inputs are important for juvenile-specific burst firing, because bath application of AMPA-type glutamate receptor antagonist (CNQX) inhibited the burst firing [27]. In addition to these neural inputs, the auto−/paracrine upregulation of firing has been suggested, based on anatomical as well as physiological experimental evidence; an electron microscopic [33] and amperometric study (Ishizaki et al., unpublished) suggested the occurrence of somatodendritic release of GnRH, single cell-RT PCR study showed the expression of GnRH receptors [34], and electrophysiological studies showed upregulation of pacemaker activities via GnRH receptors [35]. Similarly, auto−/paracrine down-regulation of firing via Neuropeptide FF (NPFF) has also been suggested. [35–38]. Thus, GnRH and NPFF are suggested to be important for regulating firing activities of TN-GnRH3 neurons in the cluster. The mechanisms of modulations by both synaptic and auto−/paracrine inputs have already been reviewed (see [13, 39]). Moreover, GnRH neurons in the terminal nerve are electrically coupled with each other [40]. The synchronization of firing by this electrical coupling and the GnRH peptide-induced upregulation (described above) is suggested to play an important role in synchronous upregulation of firing of neurons in the cluster and subsequent neuropeptide release in the broad brain area.

Various firing patterns of GnRH neurons in the terminal nerve may be related to the neuropeptide or neurotransmitter release from the GnRH neurons. It should be noted that GnRH neurons in the terminal nerve express not only neuropeptides, GnRH and NPFF, but also vesicular glutamate transporter [41], which suggests that these neurons can release glutamate, especially during low-frequency firing. In addition, a study using amperometry suggested that GnRH neurons firing at high frequency release neuropeptides [42]. These results suggest that GnRH neurons firing at high frequency during bursting release neuropeptides in addition to glutamate. Our recent study reported that GnRH neurons in the juvenile terminal nerve show spontaneous juvenile-specific burst firing (> ~ 5 Hz) and an increase in intracellular Ca^2+^ concentration [27], which is almost the same as that of hypophysiotropic GnRH1 neurons and sufficient to trigger the release of GnRH1 peptide [43, 44]. Therefore, the terminal nerve in juvenile fish is suggested to release neuropeptides more frequently than in adulthood.

#### Functions

Multiple functions of the terminal nerve have been reported recently. A study using retrograde and anterograde tracers showed that terminal nerve neurons appear to receive inputs from sensory processing neurons mainly related to olfactory, visual, and somatosensory information [25, 45]. In fact, tail pinch (noxious stimulus) decreased firing of terminal nerve neurons in goldfish [46]. Although one recent study reported that terminal nerve in zebrafish responded to CO_2_ [47], their experimental results seem to indicate that the sensory component of the trigeminal nerve, rather than the terminal nerve, may be the main sensor of CO_2_ (see ‘*GnRH neurons in the trigeminal nerve*’ section for detail). On the other hand, the application of GnRH peptide, which is thought to be released from terminal nerve, modulates neural activities of olfactory, visual, auditory processing systems, although the primary targets for GnRH neuromodulation are unclear in some cases [17–20, 48]. In addition, morphological analyses showed that the terminal nerve GnRH neurons project to the interplexiform layer of the retina, where retinal neurons express GnRH receptors [49–52]. Here, the terminal nerve GnRH neurons are suggested to modulate dopaminergic cell [50, 53] and induce the modulation of responsibility of ganglion cells to color contrast [54, 55]. These results described above suggest that terminal nerve plays a key role in the integration of multiple sensory inputs.

As for the behavioral functions, the terminal nerve has been suggested to be involved in the motivation of both male and female sexual behaviors (teleost [56–58], rodent [59]). Recently, it has been reported that the terminal nerve expresses estrogen receptor alpha, the ligand of which, estrogen, is known to be important for reproductive functions [60]. However, it is still unknown whether estrogen affects firing activities or other functions of the terminal nerve neurons, a question which will require further investigation. With recent advances in molecular genetic techniques, it has become easier to generate gene knockouts (KO) or perform genome editing, and functional studies of GnRH neurons are making rapid progresses. In zebrafish, in which *gnrh3* in wild type (WT) is expressed in both terminal nerve and preoptic area, *gnrh3* KO fish show normal development from egg to adulthood and are able to spawn normal eggs, although their sexual behaviors have not been analyzed [61]. Although studies on the functions of the terminal nerve have been performed mainly using adults, we recently reported that the terminal nerve in juvenile shows burst firing more frequently than in adulthood [27]. Because some peptidergic neurons play different roles between juvenile and adulthood [62, 63], it is possible that the terminal nerve functions differently depending on the developmental stage.

### GnRH neurons in the midbrain tegmentum

#### Anatomy

The *gnrh* gene expressed in the midbrain tegmentum is *gnrh2*, and the anatomical location of this expression is the most highly conserved among vertebrates [4, 64, 65]. However, because the mammals usually used for laboratory experiments, such as rodents and cattle, have lost *gnrh* expression in the midbrain, there have been only a few studies on the midbrain GnRH neurons to date. In this section, we summarize results from studies using teleosts and musk shrew. As for the developmental origin of GnRH neurons in the midbrain tegmentum, these neurons have been reported to arise from non-placodal origin [66, 67]. These neurons project broadly in the brain, similarly to the terminal nerve neurons, and their dense axonal projection is found in a rather caudal part of the brain (Fig. 2d) [8, 13, 68, 69]. In addition, it has been reported that they also project to the caudal neurosecretory system, which is a fish-specific organ and plays homeostatic roles in osmoregulation and reproduction [70–72]. Only one study using zebrafish reported a minor axonal projection from midbrain GnRH neurons to the pituitary, especially in fasted fish [73].

#### Physiology

The neural activities of midbrain GnRH neurons were first recorded using *gnrh2*:*gfp* medaka [22]. These neurons mainly showed intrinsic regular firing, like that reported for the terminal nerve of adult fish, and their firing frequency was ~ 1 Hz (Fig. 2e, f). Because such regular pacemaker activities are characteristic of many neuromodulatory neurons, such as aminergic (histamine, dopamine, serotonin) and peptidergic (GnRH3, orexin) neurons [74], GnRH neurons may play an important role in neuromodulatory functions.

#### Functions

The functions of midbrain GnRH neurons have mainly been analyzed by intraperitoneal injection (ip), intracerebroventricular (icv) injection, or mRNA expression analysis of GnRH2. In seabass, ip injection of GnRH2 induced an increase in melatonin release [75]. In addition, axons of the midbrain GnRH2 neurons in seabass project to the pineal organ, where the GnRH receptor is expressed [75]. Thus, the midbrain GnRH neurons appear to modulate pineal functions. On the other hand, in both goldfish and zebrafish, icv injection of GnRH2 decreased food intake [76, 77]. In addition, overfeeding induced an increase in *gnrh2* expression [77], which is suggested to be induced by α- melanocyte-stimulating hormone and corticotropin-releasing hormone (anorexigenic pathway) [78]. Icv injection of corticotropin-releasing hormone (CRH) increased *gnrh2* mRNA expression. Thus, midbrain GnRH neurons have been thought to change *gnrh2* expression according to energy balance and control food intake in teleosts [76, 77]. In musk shrew, infusion of GnRH2, but not GnRH1, into the lateral ventricle facilitated female sexual behaviors [79], and food restriction decreased *gnrh2* expression and weakened sexual behavior [80–82]. The results of these studies suggest that energy balance modulates sexual behavior through the midbrain GnRH neurons.

However, gaining a clear understanding of the functions of midbrain GnRH neurons is not so simple. Recent study reported that *gnrh2/3* double KO zebrafish can grow almost normally [61] and expresses less *agrp1*, which is one of the feeding-related genes and facilitates food intake [83, 84], compared with WT [61]. Moreover, they show an increase in *pomca* expression, which has been suggested to suppress food intake [83, 84]. There are no significant differences in the expressions of other feeding-related genes, *orexin* and *npy* (orexigenic pathway), between *gnrh2/3* double KO and WT [61]. In musk shrew, mysteriously, although food restriction increased the number of GnRH2 immunoreactive cells and the area of their fibers [79], the expression of *gnrh2* was decreased [80]. Therefore, the function of midbrain GnRH neurons in energy balance may be rather complex.

## GnRH neurons in the peripheral nervous system

### Ganglion cells of the trigeminal nerve

#### Anatomy and physiology

**Fig. 4:**
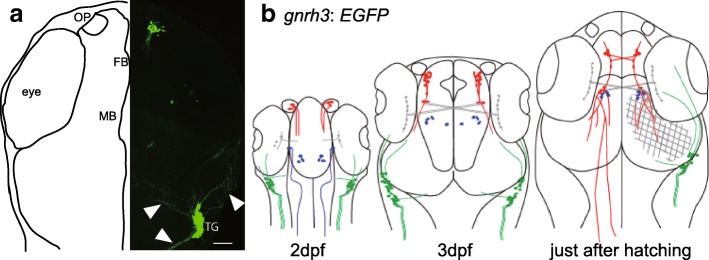
Morphology of GnRH-expressing trigeminal nerve neurons (**a**) *gnrh3*:e*gfp* neuron in medaka at 2.5 dpf. EGFP-expressing trigeminal nerves have both rostral and caudal projections (arrowhead). OP: olfactory placode, FB: forebrain, MB: midbrain, TG: trigeminal ganglion. (**b**) A schematic illustration of the projections of *gnrh3*:e*gfp* neurons at different developmental stages. Red, GnRH3 neurons in the terminal nerve and the thalamus originating from the olfactory placode, migrating to the brain, and projecting broadly in the brain. Blue, GnRH3 neurons in the thalamus originating from the brain. Because GnRH neurons in the thalamus contain only a few neurons, we did not discuss them in this review (see Takahashi et al., 2015 [9]). Green, GnRH3 neurons in the trigeminal ganglion, Gray, retinal neurons expressing EGFP ectopically. Because these ectopic retinal fiber projections and trigeminal ganglion fibers interfere with the illustration of GnRH3 neuron projections (red), we only indicated these fibers (gray and green) in the right half of the brain. During the late stages of development, red and blue neurons appeared to be intermingled. Both figures were modified from Takahashi et al., 2015 [9]

The trigeminal nerve projects broadly to the facial areas, which are roughly separated into three groups: ophthalmic, maxillary, and mandibular nerves. Some of the trigeminal ganglion cells express *gnrh*, and this expression occurs from the early developmental stages (Fig. 4) [7, 9, 26]. In teleosts, the trigeminal nerve not only projects to the facial areas [85] but also more posteriorly [9]. There are only a few reports on the firing activities of the trigeminal nerve. One study using rainbow trout, *Oncorhynchus mykiss*, reported that the trigeminal nerve did not show spontaneous action potentials so frequently, and that their firing was activated by noxious stimulation [86]. It should be noted, however, that it was unclear whether the recorded trigeminal nerve expresses *gnrh*.

#### Functions

The trigeminal nerve has been mostly studied from the medical point of view up until now. These neurons are considered to play an important role in proprioception, mechanoreception, and nociception [87]. It has been reported that trigeminal nerve components can induce oculocardiac reflex, although their relation to the vagus nerve is not known [88]. This reflex has been shown to decrease heart rate through acetylcholine release from the vagus nerve by stimulating the trigeminal nerve, especially the ophthalmic nerve. This response is hypothesized to be a possible mechanism underlying sudden infant death syndrome, and has recently been attracting scientific attention. In teleosts, the trigeminal nerve is one of the most important pathways for nociception [89]. Subcutaneous injection of acid and heat injury of skin facilitated the firing of the trigeminal nerve [90]. A recent study using transgenic zebrafish whose trigeminal nerve are labeled with GCaMP (genetic Ca^2+^ indicator) reported that CO_2_ increased intracellular Ca^2+^ concentration of the trigeminal nerve [47]. Because ablation of nose or trigeminal ganglion diminished CO_2_-induced behavior, the trigeminal ganglion, which projects to the nasal aperture, was concluded to be important for such behavior. Since the laser photoablation of the terminal nerve neurons affected CO_2_-induced slow avoidance behavior, they further concluded that the terminal nerve neurons functions to trigger such behavior as a CO_2_ sensor. However, because the terminal nerve neurons in larva are located near the sensory component of the trigeminal nerve in the nasal region, laser photoablation of the terminal nerve neurons may have damaged it. Thus, the sensory component of the trigeminal nerve, probably not the terminal nerve, is thought to play a pivotal role in triggering a slow chemosensory avoidance behavior in the larval zebrafish. In any case, as described in this section, the trigeminal nerve has various functions, and it is therefore necessary to determine which function(s) are directly related to the GnRH-expressing neurons in the trigeminal nerve in the future.

### Sympathetic preganglionic neurons

#### Anatomy

Sympathetic nerve ganglions are located along the spinal cord and receive projections from the sympathetic preganglionic neurons in the spinal cord through the rami communicantes [91]. Jan et al. [92] reported that the terminals of the sympathetic preganglionic nerve were GnRH-immunoreactive. Troskie et al. [93] reported that GnRH2 (so called “chicken GnRH II”) may play a role in the synaptic transmission in the sympathetic ganglia via a receptor specific for GnRH2. However, the origins of the GnRH-immunoreactive fibers remained unclear. While the midbrain GnRH neurons send massive axonal projections to the spinal cord (see also ‘*GnRH neurons in the midbrain tegmentum*’ section), they do not project beyond the spinal cord [8, 68, 69, 73]. Interestingly, a recent study of transgenic medaka suggested that interneurons in the spinal cord express *gnrh2*. Kusakabe et al. [69] generated transgenic medaka, in which GFP-expression is driven by noncoding sequence (5-kb upstream region of *gnrh2*) conserved among teleosts. They suggested from morphological observation that *gnrh2*:*egfp* is expressed by at least two types of neurons, the commissural interneurons and a group of ipsilaterally-projecting interneurons. In addition, larvae of *gnrh2*:*egfp* tunicate also expressed GFP in the caudal nerve cord and the motor ganglia under the control of the *gnrh2 cis*-regulatory region [69]. From these observations, they suggest a possible function of *gnrh2* neurons in the spinal cord as a component in the regulation of locomotion and swimming behavior. However, considering the previous literature since 1970s on the physiological mechanism of GnRH-immunoreactive preganglionic fiber to the sympathetic ganglion cells (see below), it is mysterious that GnRH neurons are involved in the central pattern generator circuit. Thus, it is necessary to investigate the origin and functions of GnRH-immunoreactive fibers in the spinal cord more precisely.

#### Physiology

Sympathetic ganglionic cells and sympathetic preganglionic nerve have been used as a valuable model for the analyses on both fast and slow cholinergic neurotransmission. Since the 1930s, it has been generally accepted that acetylcholine is the excitatory transmitter contained in preganglionic fibers of the sympathetic ganglia. For the following three decades, the cellular mechanisms of the cholinergic neurotransmission were studied, and it was found that the fast neurotransmission results from the action of the acetylcholine on curare-sensitive nicotinic receptors and the transient opening of cation-selective ionic channels triggered by acetylcholine [94, 95]. On the other hand, the slow component is generated by increases in Na^+^ and Ca^2+^ conductance following muscarinic acetylcholine receptor activations [96]. In 1979, the generation of the late slow excitatory postsynaptic potential (EPSP), originally called the late afterdischarge [97], was characterized as the function of GnRH [16]. This was the first report of peptidergic neuromodulation, and the actual release of GnRH from the spinal cord was suggested by radioimmunoassay and immunohistochemistry. In addition, application of GnRH suppressed M current (K^+^ current that is inhibited by muscarinic acetylcholine receptor activation) and facilitated the excitability of sympathetic ganglion cells [92, 98]. These studies formed the basis for the concept of peptidergic neuromodulation—‘neuropeptide action at a distance’ [99]. It has been reported that GnRH is released from axon terminals of the sympathetic preganglionic cells undergoing high frequency firing and an increase in the intracellular Ca^2+^ concentration [100, 101]. Subsequently, GnRH-induced modulation of Ca^2+^ conductance in addition to K^+^ one was reported; GnRH partially reduced Ca^2+^ (high voltage activated, HVA) currents [102], which also occurred in TN-GnRH3 neurons of a teleost brain [36]. Here, GnRH was suggested to shift the state of HVA channel into a “reluctant” gating mode, where opening requires stronger depolarization. This mechanism may enable presynaptic neuron (in this case, sympathetic ganglion cells) to release neurotransmitter during bursts of high frequency activity [102]. The GnRH-induced modulations of ionic currents in the sympathetic ganglion cells described thus far are suggested to coordinate an interaction between vasomotor and exocrine outflow in the sympathetic nervous system [103, 104]. Such neuromodulatory actions of GnRH may occur in the central nervous system as well [105]. Since the GnRH receptor activation mechanisms are considered to be similar among different GnRH subtypes [14, 15], the neuromodulatory actions of GnRH discussed in this section may also apply to the TN-GnRH3 system as well.

## Conclusions

Non-hypophysiotropic GnRH neurons in the terminal nerve and the midbrain tegmentum have broad axonal projections in the brain and show pacemaker activities in the adulthood. These characters are not specific to GnRH neurons, but rather common to other aminergic/peptidergic modulatory neurons. Furthermore, neurons in the various brain regions also express GnRH receptors [106, 107]. Because all GnRH subtypes can activate GnRH receptors regardless of subtypes [14, 15], it has been suggested that non-hypophysiotropic GnRH neurons in the brain also function as neuromodulators, as has been shown for the sympathetic preganglionic neuron.

Recent techniques, such as transgenic or knockout animals, have advanced our understanding of the function of non-hypophysiotropic GnRH neurons (Table 1). As discussed in the present article, it has become clear that their functions are not necessarily related to reproduction, unlike the hypophysiotropic GnRH neurons. Rather, they show neuromodulatory functions regulating behavioral motivation, food intake, sensory reception, etc. Thus, we may conclude that they function as a more general regulator of neural circuits, depending on the internal condition or the external environment. On the other hand, we have recently reported that the terminal nerve neurons in juvenile fish show more active firing than in adulthood [27]. Because it has been reported that some neuropeptides play a neurotrophic role in juvenile animals [62, 63], it is possible that neuropeptide(s) released from the terminal nerve neurons may also have a neurotrophic function. Thus, we here propose that we should broaden our concept of neuromodulation to include such neurotrophic function in juveniles in addition to the so-called neuromodulatory functions in adulthood as described above. As has already been argued previously [4, 13], non-hypophysiotropic as well as hypophysiotropic GnRH neurons are evolutionally conserved among vertebrates. Interestingly, recent studies reported that modulatory neurons [108–110], in addition to TN-GnRH3 neurons [41] and hypophysiotropic GnRH neurons [111], also release classical neurotransmitters, GABA or glutamate. Thus, it is being generally accepted that peptidergic neurons release co-transmitter(s). Our future research will seek to clarify the actual timing and functions of the non-hypophysiotropic release of GnRH and co-transmitter in vivo and to understand the significance of the fact that non-hypophysiotropic neurons projecting to broad brain regions release GnRH in addition to other co-transmitter(s).

**Table 1:** Proposed functions of GnRH-expressing neurons in literature

Neuron type	Function	References
Terminal nerve	Nociception	[46]
	Female sexual behavior	[58]
	Male sexual behavior	[56, 59]
Midbrain tegmentum	Food intake	[76]
	Reproductive energy balance	[80]
Ganglion cells of the trigeminal nerve	Nociception/ Proprioception/Mechanoreception	[87]
	CO_2_ sensor	[47]
Sympathetic preganglionic cells in the spinal cord	Interaction between vasomotor and exocrine outflow in the sympathetic nervous system	[104]

## Data Availability

Not applicable.
